# The Impact of Serum Glucose, Anti-Diabetic Agents, and Statin Usage in Non-small Cell Lung Cancer Patients Treated With Definitive Chemoradiation

**DOI:** 10.3389/fonc.2018.00281

**Published:** 2018-07-27

**Authors:** Nick A. Iarrobino, Beant S. Gill, Mark Bernard, Rainer J. Klement, Maria Werner-Wasik, Colin E. Champ

**Affiliations:** ^1^School of Medicine, University of Pittsburgh School of Medicine, Pittsburgh, PA, United States; ^2^Department of Radiation Oncology, University of Pittsburgh Medical Center, Pittsburgh, PA, United States; ^3^Department of Radiation Medicine, University of Kentucky, Lexington, KY, United States; ^4^Department of Radiation Oncology, Leopoldina Hospital, Schweinfurt, Germany; ^5^Department of Radiation Oncology, Thomas Jefferson University Hospital, Philadelphia, PA, United States

**Keywords:** lung cancer, blood glucose, chemotherapy, radiation therapy, metformin, statins, tumor metabolism

## Abstract

**Introduction:** Epidemiologic data indicate diabetes confers an augmented risk of lung cancer development, yet the relationship between hyperglycemia, metabolic agents, and prognosis is unclear. We analyzed the impact of hyperglycemia, anti-diabetic agents, and statins on outcomes in non-small cell lung cancer (NSCLC) patients undergoing chemoradiation.

**Method and Materials:** In total, data from 170 patients with stage III NSCLC treated at the University of Pittsburgh Medical Center between 2001 and 2014 were obtained for analysis. Kaplan-Meier survival analysis was used to estimate time-to-event for overall survival (OS), disease-free survival, distant metastasis (DM), and loco-regional control (LRC). Blood glucose values (*n* = 2870), statins, and diabetic medications were assessed both continuously and categorically in univariable and multivariable Cox proportional hazard regression models to estimate hazard ratios and identify prognostic factors.

**Results:** Tumor volume was a negative prognostic factor for OS, disease-free survival, DM, and LRC (*p* = 0.001). Tumor stage and treatment time were associated with increased all-cause mortality. Any glucose measurement ≥ 130 mg/dl during treatment (2-year estimate 49.9 vs. 65.8%, *p* = 0.095) was borderline significant for decreased LRC, with similar trends on multivariable analysis (HR 1.636, *p* = 0.126) and for OS (HR 1.476, *p* = 0.130). Statin usage was associated with improved 2-year LRC (53.4 vs. 62.4%, *p* = 0.088) but not with improvements in survival. Other glycemic parameters, comorbid diabetes diagnosis, or anti-diabetic medications were not significantly associated with outcomes.

**Conclusions:** There were trends for blood glucose value over 130 mg/dl and statin nonuse being associated with inferior prognosis for LRC in stage III NSCLC patients; glycemic state, statin usage, and glucose-modulating medications were not associated with survival outcomes in multivariable analysis in this retrospective database.

## Introduction

Lung cancer is the leading cause of cancer mortality globally (1). While non-small cell lung cancer (NSCLC) comprises 85% of lung cancer cases in the United States, survival rates remain dismal. The 5-year overall survival rate for NSCLC is 21% (2), illustrating the need for better treatment options.

Diabetes has been implicated as a risk factor for cancer development at multiple sites including the pancreas, esophagus, liver, colon, and breast (3, 4). Insulin, a mitogenic hormone, is elevated in the setting of type 2 diabetes. By both binding its receptor and increasing bioavailability of insulin-like growth factor 1 (IGF-1), these peptides are free to exhibit potent anti-apoptotic and cell proliferative effects. Additionally, given glucose's efficacy in stimulating expression of the insulin receptor (IR) as well as modulating its affinity for insulin, hyperglycemia, and hyperinsulinemia are speculated to promote cancer development and proliferation via activation of IR and downstream provocation of the PI3K-Akt-mTORC1 and MAPK pathways (5, 6) Furthermore, proliferation of lung cancer cells correlates with uptake of the glucose-based tracer [F-18] fluorodeoxyglucose during positron emission tomography (PET) scans (7). Yet, it remains unclear if an elevated blood glucose could enhance tumor metabolism and affect clinical outcomes in this manner.

Hyperglycemia has reliably been associated with a poorer prognosis in other cancers, yet the effect of hyperglycemia on survival in lung cancer patients undergoing definitive treatment with chemoradiation is unknown (8, 9). To our knowledge, only three studies have described the effect of fasting serum glucose on survival in NSCLC; all found elevated serum glucose levels to be associated with diminished overall survival (10–12). However, other studies have reported that diabetes-induced microangiopathy may protect vascular basal membranes from tumor cell digestion, thus interfering with neoplastic cell spread and improving survival (13).

Other data implicate the utilization of cholesterol for cancer metabolism, exemplified by associations between low serum cholesterol and protection from cancer development (14). Statins, which inhibit 3-hydroxy-3-methylglutaryl coenzyme A (HMG-CoA) reductase, exhibit pleotropic effects. Experimental studies have demonstrated cytotoxic effects in cancer cells stemming from increased intracellular reactive oxygen species (ROS) production (15), altered protein expression (16, 17), and potential radiosensitizing effects (18). Simvastatin has been shown to decrease Bcl-2 expression, increase Bax protein expression, and halt G_1_-S cell cycle progression in human lung cancer cells in a dose-dependent manner (19). Recent studies have revealed evidence for enhanced survival in statin users at various sites including prostate (20), colorectal (21), and breast cancer (22). We previously reported improved mortality and distant metastases in advanced-stage pancreatic cancer patients using statin medications (23).

The role hyperglycemia, antidiabetics, and statins play in lung cancer prognosis has not been fully elucidated. In this study, we examine the effect of serum glucose levels before, during, and after treatment, along with statins and anti-diabetic medication exposure on overall survival, disease-free survival, distant metastasis, and loco-regional control in patients with stage III NSCLC treated with definitive chemoradiation.

## Materials and methods

### Patient population

After institutional review board approval, we retrospectively evaluated the medical records of all patients diagnosed with stage III NSCLC, determined to have unresectable disease, and treated with definitive chemoradiation with curative intent at the University of Pittsburgh Medical Center between 2001 and 2014. Tumor staging was conducted in accordance with the American Joint Committee on Cancer (AJCC) seventh edition. Patients received daily radiotherapy treatment concurrently with platinum-based doublet chemotherapy.

### Measures

Patient characteristics and clinical laboratory values were obtained from hospital records; including age, race, gender, pre-radiotherapy body mass index (BMI), comorbidities, medications, and chemotherapeutic agents. All serum glucose values available in the electronic medical record were initially collected. These values were non-fasting, random blood draws. In many cases glucose was assessed at numerous time points during the day and patients were therefore in differing glycemic states. To reduce the impact of this bias, we included all glucose values (*n* = 2870) spanning from 90 days prior to 90 days post chemoradiation for analysis. We then grouped these values into time points 1, 2, and 3, defined as within 90 days prior to radiotherapy, during radiotherapy, and within 90 days post-radiotherapy, respectively. Maximum, median, and minimum serum glucose values at the preceding time points were analyzed as continuous variables in univariable analysis with respect to overall survival, disease-free survival, distant metastasis, and loco-regional control.

Additionally, at each time point we stratified the maximum blood glucose value achieved into four levels: ≥130 mg/dl, ≥150 mg/dl, ≥175 mg/dl, and ≥200 mg/dl. Values were selected based on normal lab cutoff values, previous results (10), and to provide ample data points for thorough analysis. Univariable Cox regression was again conducted to assess the effect maximum blood glucose value has on our primary outcomes of interest. Serum blood glucose at the aforementioned time points was considered the main prognostic factor of interest.

Prior literature has recommended the use of a lag period in observational cancer studies (24), after which patients switch from the non-exposed to exposed group. Similar reports have found a lag period of 6 months to be an appropriate time span (21), and to mitigate risk of reverse-causation bias, we considered patients in the statin exposure group after 6 months of continuous statin therapy. Previous studies have collected cumulative statin dose in order to evaluate the prospect of a dose-response relationship between dose and cancer-specific outcomes (25). Furthermore, earlier investigations have explored various pharmacologic properties of individual statin agents, namely potency and lipophilicity status, as potential factors influencing clinical outcomes at other sites (26). To evaluate these potential relationships in NSCLC patients we collected simvastatin and atorvastatin dosages, standardized to simvastatin equivalents, and stratified dosages into intensity interval groups. However, as simvastatin was the predominant prescription agent, and statin doses were sporadically recorded, they were excluded from analysis, and stratification of therapeutic agents for individual analysis was omitted.

### Statistical analysis

Overall survival, disease-free survival, freedom from distant metastasis, and loco-regional control were the primary outcomes measured. Loco-regional control was defined as the absence of disease progression seen on follow-up imaging or primary and/or regional lymph node biopsy. Kaplan-Meier survival analysis was conducted to estimate the actuarial event probability for each outcome. Select variables were assessed, including patient characteristics, comorbidities, medications, tumor characteristics, radiation therapy planning details, and treatment time. Those achieving or approaching statistical significance on univariable survival analysis (*p* < 0.10) were considered for multivariable Cox regression while additionally including any glucose measurement ≥130 during RT and glucocorticoid use as main variables of interest. In addition, given recent findings that glucocorticoids increase radioresistance in glioblastoma cells and given their property to elevate blood glucose levels, we included glucocorticoid usage in all multivariable models besides a measure of blood glucose (27, 28). Hazard ratios were then calculated to determine the magnitude of the effect. *P*-values < 0.05 and < 0.1 were considered as statistically “significant” and “trending”, respectively. All statistical analyses were done using IBM SPSS Statistics Version 23 (IBM, Armonk, NY).

## Results

### Patient characteristics

We identified 576 patients with newly diagnosed stage IIIA or IIIB NSCLC, among which those treated surgically (*n* = 28) or with palliative intent (*n* = 296) were excluded. Patients receiving radiation therapy without concurrent chemotherapy (*n* = 39) were also excluded. Of the remaining 213 patient cohort, 43 patients were lost to follow up directly after treatment, leaving 170 patients. Our study cohort was composed of 110 males (64.7%) and 60 females (35.3%). The median age of subjects included for analysis was 67 years. The majority of patients were diagnosed with stage IIIA NSCLC (57.6%) and received chemotherapy as carboplatin-paclitaxel (87.6%). Associated major comorbid states in this cohort were as follows: diabetes mellitus (28.2%), coronary artery disease (11.2%), hyperlipidemia (31.8%), and hypertension (44.7%). Comorbidity prevalence in this cohort was comparable to a recent population based study reporting major comorbidity prevalence in the United States (29). A detailed description of patient, disease, and treatment characteristics is presented in Table 1.

**Table 1:** Patient, disease and treatment characteristics (*n* = 170).

**PATIENT CHARACTERISTICS**	
Age, years Median (range)	67 (38–91)
**GENDER**	
Male	110 (64.7%)
Female	60 (35.3%)
**Race**	
Caucasian	137 (80.6%)
African American	33 (19.4%)
**Body mass index**	
Median (range)	26.2 (16.0–55.1)
Normal	33%
High	62%
**Comorbidities**	
Diabetes	48 (28.2%)
Coronary artery disease	19 (11.2%)
Hyperlipidemia	54 (31.8%)
Hypertension	76 (44.7%)
**Medications taken at the time of treatment**	
Metformin	9 (5.3%)
Sulfonylurea	9 (5.3%)
Insulin	44 (25.9%)
Statin	53 (31.2%)
Glucocorticoid	72 (42.4%)
**DISEASE CHARACTERISTICS**	
**Histology**	
Squamous cell	54 (31.8%)
Adenocarcinoma	77 (45.3%)
Large cell neuroendocrine	10 (5.9%)
Mixed	2 (1.2%)
Not otherwise specified	27 (15.9%)
**T stage**	
T0	16 (9.4%)
T1	32 (18.8%)
T2	53 (31.2%)
T3	26 (15.3%)
T4	43 (25.3%)
**N stage**	
N0	10 (5.9%)
N1	6 (3.5%)
N2	102 (60.0%)
N3	52 (30.6%)
**AJCC stage (7th edition)**	
IIIA	98 (57.6%)
IIIB	72 (42.4%)
**TREATMENT CHARACTERISTICS**	
**Chemotherapy regimen**	
Carboplatin/paclitaxel	149 (87.6%)
Cisplatin/etoposide	5 (2.9%)
Carboplatin/pemetrexed	2 (1.2%)
Carboplatin/etoposide	5 (2.9%)
Carboplatin/docetaxel	3 (1.8%)
Cisplatin/docetaxel	3 (1.8%)
Carboplatin/nab-paclitaxel	1 (0.6%)
Cisplatin/gemcitabine	1 (0.6%)
Unknown	1 (0.6%)
**Radiation technique**	
3D conformal	117 (68.8%)
IMRT	48 (28.2%)
Unknown	5 (2.9%)
**GTV volume (cc)**	
Median (range)	84.7 (4.4–586.4)
**PTV volume (cc)**	
Median (range)	340.0 (43.0–1303.0)
**Cumulative radiation dose (Gy)**	
Median (IQ range)	72 (68.4–77.3)
**Dose per fraction (Gy/fraction)**	
Median (IQ range)	2.0 (2.0–2.1)
**Number of elapsed days for radiation treatment**	
Median (IQ range)	52 (49–56)

Median blood glucose values per patient did not substantially change throughout the course of treatment. Among individual patient median glucose values, the median measurements within 90 days prior to radiation, during radiation, and within 90 days post-radiation therapy were 106.8 mg/dl, 106.0 mg/dl, and 111.0 mg/dl, respectively (Table 2).

**Table 2:** Glucose values (*n* = 2870) measured prior to, during and after chemoradiation.

	**Within 90 days prior to radiation**	**During radiation**	**Within 90 days after radiation**
**Number of glucose measurements per patient**			
Median (range), mg/dL	2 (0–67)	6 (0–43)	3 (0–124)
**Median glucose measurement per patient**			
Median (range), mg/dL	106.8 (52.5–269.0)	106.0 (79.0–317.0)	111.0 (63.0–241.9)
**Minimum glucose measurement per patient**			
Median (range), mg/dL	95.0 (47.0–168.0)	91.0 (57.0–237.0)	94.5 (36.0–172.0)
**Maximum glucose measurement per patient**			
Median (range), mg/dL	120.5 (81.0–500.0)	132.0 (88.0–355.0)	138.0 (82.0–469.0)
**Number of patients with any glucose value:**			
≥130 mg/dL, *n*(%)	50 (29.4%)	60 (35.3%)	58 (34.1%)
≥150 mg/dL, *n*(%)	32 (18.8%)	50 (29.4%)	49 (28.8%)
≥175 mg/dL, *n*(%)	23 (13.5%)	30 (17.6%)	35 (20.6%)
≥200 mg/dL, *n*(%)	18 (10.6%)	17 (10.0%)	26 (15.3%)

### Univariable analyses

**Figure 1:**
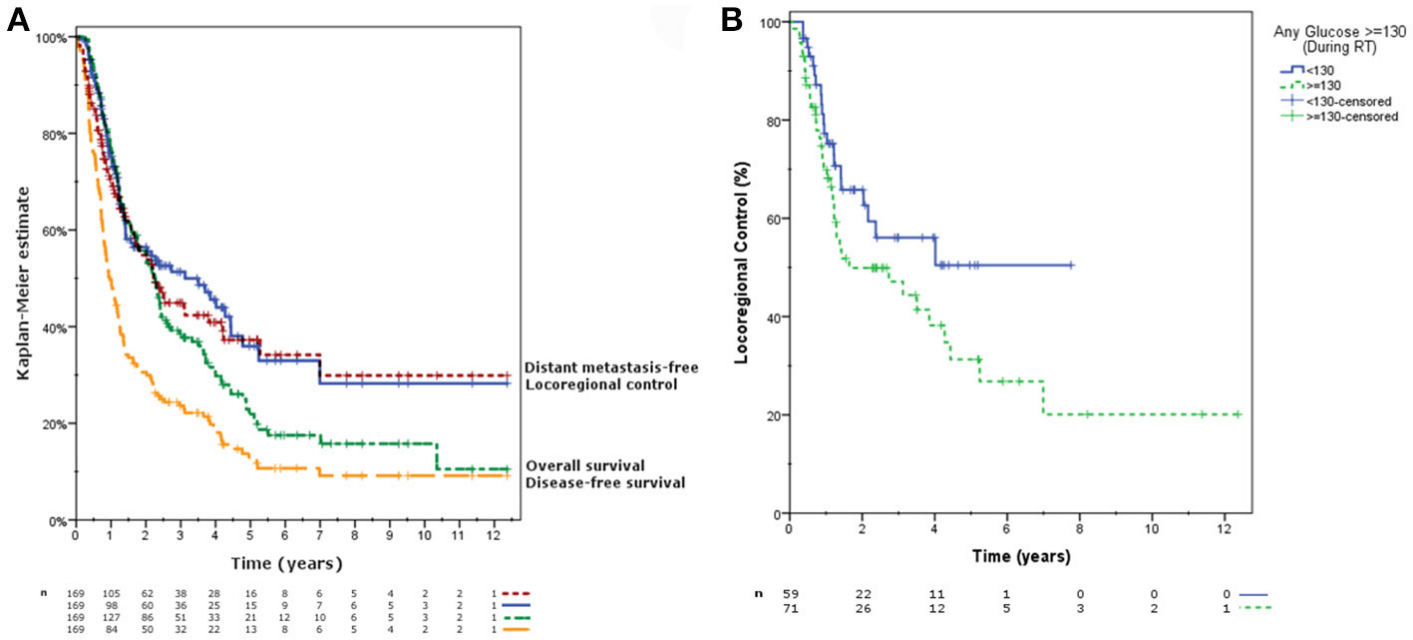
**(A)** Kaplan-Meier estimated locoregional control, distant metastasis rate, and overall survival for all patients. **(B)** Kaplan-Meier estimates of locoregional control between groups with any serum glucose reading below or ≥130 mg/dL during the course of radiotherapy (*p* = 0.095).

With a median follow up time of 24.2 months (range, 3.2–148.4), the 2-year Kaplan-Meier estimates were as follows: locoregional control 56.5% (95% CI 48.3–64.7%), distant metastasis rate 54.8% (95% CI 46.4–63.2%), disease-free survival 30.6% (95% CI 23.5–37.7%), and overall survival 55.1% (95% CI, 47.5–62.7%) (Figure 1A).

Gross tumor volume (GTV) and planning target volume (PTV) were consistent significant predictors associated with all outcomes as continuous variables (Table 3). Patients with any blood glucose value ≥130 mg/dL during treatment trended toward worse locoregional control (2-year estimate 49.9 vs. 65.8%, *p* = 0.095) (Figure 1B). No other glucose parameters (median, minimum, maximum, or any value with variable cut-offs) were significantly associated with outcomes. Other significant associations included prolonged treatment time with inferior overall survival (unadjusted HR 1.020, *p* = 0.044), stage IIIB disease with inferior disease-free survival (43.1 vs. 63.6%, *p* = 0.021) and overall survival (26.4 vs. 33.6%, *p* = 0.085), and glucocorticoid usage during treatment with improved overall survival (59.1 vs. 51.4%, *p* = 0.056). No other glucose-modulating medications were significantly associated with outcomes.

**Table 3:** Univariable survival analysis for various endpoints *(Kaplan Meier survival differences and/or Cox regression hazard ratios included for significant or borderline significant results)*.

	**Locoregional control *p* (2y estimate/HR)**	**Distant metastasis *p* (2y estimate/HR)**	**Overall survival *p* (2y estimate/HR)**	**Disease-free survival *p* (2y estimate/HR)**
Age (continuous)	0.642	0.477	0.609	0.811
Gender	0.139	0.780	0.424	0.486
Race	0.797	0.549	0.813	0.697
BMI (continuous)	0.136	0.269	0.780	0.543
**Comorbidities**				
Diabetes	0.132	0.833	0.808	0.650
Coronary artery disease	0.257	0.523	0.706	0.772
Hyperlipidemia	0.180	0.509	0.337	0.671
Hypertension	0.532	0.847	0.807	0.434
**Medications**				
Metformin	0.293	0.650	0.206	0.808
Sulfonylurea	0.182	0.711	0.406	0.653
Insulin	0.171	0.650	0.738	0.851
Statin	**0.088 (53.4 vs. 62.4%)**	0.448	0.673	0.636
Glucocorticoid	0.200	0.944	**0.056 (51.4 vs. 59.1%)**	0.297
Histology	0.668	0.561	0.720	0.425
Stage	0.929	0.105	**0.021 (63.6 vs. 43.1%)**	**0.085 (33.6 vs. 26.4%)**
Radiation Technique	0.778	0.674	0.319	0.895
GTV volume (continuous)	**0.006 (HR 1.004)**	**0.012 (HR 1.003)**	**0.002 (HR 1.003)**	**0.001 (HR 1.003)**
PTV volume (continuous)	**0.003 (HR 1.002)**	<**0.001 (HR 1.002)**	<**0.001 (HR 1.002)**	<**0.001 (HR 1.002)**
Cumulative radiation dose (continuous)	0.826	0.534	0.781	0.734
Number of elapsed days on treatment (continuous)	0.539	0.893	**0.044 (HR 1.020)**	0.447
**Median glucose value (continuous)**				
Pre-treatment	0.397	0.541	0.463	0.392
During treatment	0.488	0.691	0.593	0.977
Post-treatment	0.317	0.520	0.346	0.762
**Minimum glucose value (continuous)**				
Pre-treatment	0.480	0.206	0.366	0.494
During treatment	0.394	0.506	0.745	0.616
Post-treatment	0.161	0.686	0.296	0.676
**Maximum glucose value (continuous)**				
Pre-treatment	0.369	0.842	0.297	0.306
During treatment	0.940	0.675	0.331	0.459
Post-treatment	0.819	0.498	0.530	0.821
**Any pre-treatment glucose value**				
≥130 mg/dL	0.630	0.874	0.847	0.734
≥150 mg/dL	0.443	0.974	0.757	0.531
≥175 mg/dL	0.110	0.765	0.168	0.156
≥200 mg/dL	0.234	0.930	0.338	0.306
**Any treatment glucose value**				
≥130 mg/dL	**0.095 (49.9 vs. 65.8%)**	0.493	0.939	0.840
≥150 mg/dL	0.643	0.666	0.595	0.797
≥175 mg/dL	0.321	0.877	0.396	0.570
≥200 mg/dL	0.386	0.518	0.918	0.878
**Any post-treatment glucose value**				
≥130 mg/dL	0.958	0.390	0.274	0.518
≥150 mg/dL	0.829	0.451	0.138	0.560
≥175 mg/dL	0.564	0.911	0.976	0.506
≥200 mg/dL	0.704	0.680	0.920	0.750

*Bold values statistically significant*.

In unadjusted analysis (Table 3), baseline statin use was not associated with enhanced overall survival (*p* = 0.673), freedom from distant metastasis (*p* = 0.448), or disease-free survival (*p* = 0.636). Baseline statin usage trended with improved 2-year locoregional control (53.4 vs. 62.4%, *p* = 0.088).

### Multivariable analyses

Significant variables from univariable analysis plus the main variables of interest were subjected to multivariable Cox regression to identify independent prognostic factors. After controlling for other potential confounding variables, increasing PTV volume was correlated with inferior outcomes for all endpoints (Table 4). Having any glucose measurement ≥130 mg/dL during chemoradiotherapy was associated with inferior outcomes, reaching borderline significance for locoregional control (adjusted HR 1.636, *p* = 0.126) and overall survival (adjusted HR 1.476, *p* = 0.130).

**Table 4:** Multivariable Cox regression survival analyses using forward conditional analysis (*p* < 0.10 for model inclusion).

**Variable**	**Adjusted HR (95% CI)**	***p***
**LOCOREGIONAL CONTROL**		
PTV volume (cc)	1.002 (1.001–1.003)	**0.002**
Glucose measurement ≥130 during RT	1.636 (0.871–3.070)	0.126
Glucocorticoid use	0.786 (0.430–1.436)	0.434
Statin use	0.806 (0.423–1.536)	0.512
**DISTANT METASTASIS-FREE**		
PTV volume (cc)	1.002 (1.001–1.003)	<**0.001**
Glucose measurement ≥130 during RT	1.301 (0.739–2.289)	0.362
Glucocorticoid use	1.000 (0.573–1.746)	1.000
**Stage**		
IIIA	1.000 (Reference)	
IIIB	0.722 (0.371–1.406)	0.338
**OVERALL SURVIVAL**		
PTV volume (cc)	1.001 (1.000–1.002)	**0.007**
Glucose measurement ≥130 during RT	1.476 (0.892–2.442)	0.130
Glucocorticoid use	0.640 (0.385–1.063)	0.085
**Stage**		
IIIA	1.000 (Reference)	
IIIB	1.460 (0.846–2.519)	0.174
Treatment duration (days)	1.019 (0.988–1.050)	0.229
Radiation dose (Gy)	0.966 (0.921–1.013)	0.155
**DISEASE-FREE SURVIVAL**		
PTV volume (cc)	1.002 (1.001–1.003)	<**0.001**
Glucose measurement ≥130 during RT	1.112 (0.706–1.750)	0.646
Glucocorticoid use	0.890 (0.569–1.391)	0.608
**Stage**		
IIIA	1.000 (Reference)	
IIIB	1.297 (0.802–2.096)	0.289

*Bold values statistically significant*.

## Discussion

In this retrospective-cohort study, we assessed the effect blood glucose, anti-diabetics, and statin medications may have on outcomes in newly diagnosed stage III NSCLC patients. Using our retrospective data set with an extensive number of glucose values, we observed that a glucose value ≥ 130 mg/dL trended with diminished locoregional control and overall survival, while statin usage trended with improved locoregional control. We were otherwise unable to establish a substantial relationship between blood glucose, anti-diabetic medication, or statin use and any of the aforementioned time points and cancer-specific outcomes. To our knowledge, this is the largest study to explore these interactions in a strict NSCLC cohort.

As expected, tumor stage and size were significant prognostic factors, and prolonged treatment time was associated with increased mortality, all of which have been shown to correlate with poorer outcomes previously (30, 31). In addition, contrary to other data, body mass index was not associated with prognosis in this cohort (32). Although our data suggest that glucocorticoid usage at or before diagnosis may be associated with improved overall survival, this was not supported in multivariable analysis and may have simply been an artifact of the data.

A growing body of literature suggests that hyperglycemia may behave as a prognostic factor in cancer outcomes, including in cancer sites like high-grade gliomas, which has been inversely proportional to serum glucose in multiple studies (33–36). Early investigations exploring the relationship between diabetes and overall survival in NSCLC reported longer survival times in patients with diabetes (37), based on work suggesting that diabetic microangiopathy may prevent the spread of tumor cells (13). More recent literature yields evidence for positive (38), negative (10, 39), and non-significant negative associations (40) between diabetes and survival. We observed no relationship between diabetes and overall survival nor distant metastasis, suggesting that there is no clear evidence to support microangopathy as a contributory factor to protection against degradative tumor enzymes and distant metastasis.

Preclinical data demonstrates that inhibition of PI3K (41), Akt (42), and mTOR (43) via molecular antagonists reduces tumor growth and proliferation in murine models. Hyperglycemia enhances WNT/β-catenin signaling in tumor cells (44), which is associated with chemo- and radiotherapy resistance in NSCLC (45). While preclinical data suggest that cancer treatment may be enhanced via inhibiting these proteins with antagonist molecules, downregulation by means of intense lifestyle changes, dietary alterations, or modulation of insulin/glucose through diabetic medication remains less clear in practice (5, 6). In other sites blood glucose and/or insulin reduction via metformin, or a ketogenic diet is being attempted to potentially offset these glucose-fueled pathways (46–49). Still, preclinical data reveal that tumors with PI3K activation may be resistant to dietary restriction (50). While metformin usage has been associated with improvements in survival in advanced stage lung cancer patients, our data revealed no apparent benefit (51).

In contrast to earlier findings (10), we did not observe a relationship between serum glucose and survival after comprehensive statistical analysis, except for borderline significance in univariable analysis. The reasons for this are less clear, however, Luo et al. noted that 82.8% of their study population consisted of advanced stage (stage IIIB or IV) NSCLC patients in comparison to our 42.4% stage IIIB population. Advanced stage disease could correlate with insulin resistance and hence increased blood glucose, a phenomena of metabolic dysregulation and cachexia that is not uncommon in these patients (52). Another possible explanation for these differing results may be a lack of adequate data points in the preceding study, with the present study assessing 2870 glucose values versus 342 in the other. Moreover, the above study also failed to account for glucocorticoid usage, which would be required in patients presenting with severe disease symptoms, thus offering a considerable confounding factor.

Data on the benefit of statins in improving outcomes in lung cancer patients remain mixed. Similar to our study, a recent phase II clinical trial in 106 advanced-stage NSCLC (stage IIIB or IV) revealed a weak, non-significant survival improvement in a group that received gefitinib plus simvastatin compared to gefitinib alone (53). A retrospective study revealed survival benefits from statin usage in epidermal growth factor positive NSCLC, however, they did not account for reverse-causation bias (54). Another population-based cohort study noted a 12% reduction in the rate of lung-cancer specific mortality in NSCLC and small cell cancer patients using statins before diagnosis, including a trend in the reduction of lung-cancer specific mortality with statin use after diagnosis (55).

Small cell lung cancer malignancies are significantly more radiosensitive, and statins impede progression from G_1_-S in the cell cycle, potentially explaining the discrepancy between results (56). On a cellular level, these changes would confer a potential radiosensitizing effect to act synergistically with local treatment and potentially enhance radiation response. Furthermore, statins can mimic mutant p53 depletion and arrest cells in G1 by halting cell cycle progression, both of which may enhance radiosensitizing effects (57–59).

We cannot infer whether these mechanisms would influence our results as well, but the finding that statin usage was associated with improved locoregional control (*p* = 0.088 in univariable analysis) would be consistent with them.

Inherent to observational studies, the risk of both confounding and reverse-causation bias is present in our study. By utilizing a 6-month lag period we have attempted to alleviate some risk of reverse-causation producing erroneous results. As with all observational studies, and even randomized controlled trials (60), eliminating potential confounders in their entirety is not possible. Nonetheless we attempted to control for putative prognostic factors by including all variables achieving or approaching significance at the *p* = 0.10 level in multivariable Cox regression in addition to the main variables of interest (hyperglycemia and glucocorticoid usage). Statin agent dosages were seldom recorded, and we were therefore unable to establish a dose-response relationship. However, recent large analyses (25) have been unsuccessful in establishing this dose-response relationship, suggesting that statin exposure and survival may be a binary relationship, if present. Furthermore, our results may be limited by the fact that patients were not necessarily fasting when glucose labs were drawn; we attempted to mitigate this by assessing an extensive amount of glucose values around the time of treatment, totaling 2870, and representing the greatest number of data points per patient in a study of this kind. It should be noted that at least one study has shown cancer patients with a comorbid diabetes diagnosis to be treated less aggressively than their healthy counterparts (61). This potential bias is unlikely to be present as all patients in this study received similar aggressive treatment. The extensive random sampling within our patient set assessed purely serum glucose levels regardless of a diabetes diagnosis. Along these lines, the large number of glucose values and extensive statistical assessment of competing variables remains a strength of this analysis.

## Conclusion

Glucose levels, anti-diabetic medications, and statin usage were not associated with overall survival, disease-free survival, distant metastases, or loco-regional control in a robust retrospective database of NSCLC patients treated with definitive chemoradiation. While a blood glucose value over 130 mg/dl trended toward a consistent association with inferior outcomes, reaching borderline significance for locoregional control and overall survival, and statin usage trended with improved locoregional control, glycemic state, statins, and glucose-modulating medication usage was not associated with outcomes in multivariable analysis. Prognosis in NSCLC patients may be less related to metabolic factors than other cancer sites, but high quality prospective studies are further needed to elucidate the relationship between glycemic state and outcomes in NSCLC patients.

## Author contributions

NI data collection and analysis, manuscript creation and editing; BG data analysis and statistics, manuscript editing, project design; MB data collection and review, manuscript editing; RK statistics, manuscript creation and editing; MW-W manuscript editing and project design; CC data analysis, manuscript creation, and editing, project design.

### Conflict of interest statement

The authors declare that the research was conducted in the absence of any commercial or financial relationships that could be construed as a potential conflict of interest.
